# A mouse mastitis model to study the effects of the intramammary infusion of a food-grade *Lactococcus lactis* strain

**DOI:** 10.1371/journal.pone.0184218

**Published:** 2017-09-05

**Authors:** Cristina Camperio, Federica Armas, Elena Biasibetti, Paolo Frassanito, Carlo Giovannelli, Liliana Spuria, Claudia D’Agostino, Sabrina Tait, Maria Teresa Capucchio, Cinzia Marianelli

**Affiliations:** 1 Department of Food Safety and Veterinary Public Health, Istituto Superiore di Sanità, Rome, Italy; 2 Department of Veterinary Sciences, University of Turin, Turin, Italy; 3 Department of Sciences, Roma Tre University, Rome, Italy; 4 Center for Gender-Specific Medicine, Istituto Superiore di Sanità, Rome, Italy; University of Illinois, UNITED STATES

## Abstract

*Lactococcus lactis* is one of the most important microorganisms in the dairy industry and has “generally recognized as safe” (GRAS) status. *L*. *lactis* belongs to the group of lactic acid bacteria (LAB) and is encountered in a wide range of environments. Recently, the use of the intramammary infusion of a live culture of LAB has been investigated as a new antibiotic alternative for treating mastitis in dairy ruminants. Controversial results are described in literature regarding its efficacy and safety. In this study we conducted in-depth investigation of the mammary gland immune response induced by intramammary inoculum of a live culture of *L*. *lactis* LMG 7930 using the mouse mastitis model. Overnight cultures either of *L*. *lactis* (≈ 10^7^ CFU) or of the mastitis pathogens *Staphylococcus chromogenes* (≈ 10^5^ CFU) or *S*. *aureus* (≈ 10^2^ CFU/ml) were injected into the mouse inguinal glands. A double injection, consisting of *S*. *chromogenes* first and then *L*. *lactis*, was also investigated. Bacterial recovery from the gland and inflammatory cell infiltration were assessed. *L*. *lactis*-treated and control glands were analysed for proinflammatory cytokine production. Microbiological results showed that *L*. *lactis* was able to survive in the mammary gland 24 h post infection, as were the mastitis pathogens *S*. *chromogenes* and *S*. *aureus*. *L*. *lactis* reduced *S*. *chromogenes* survival in the glands and increased its own survival ability by coexisting with the pathogen. Histology showed that *L*. *lactis*-treated glands presented variable histological features, ranging from undamaged tissue with no inflammatory cell infiltrate to severe PMN infiltrate with focal areas of tissue damage. *S*. *aureus*-treated glands showed the most severe histological grade of inflammation despite the fact that the inoculum size was the smallest. In contrast, most *S*. *chromogenes*-treated glands showed normal structures with no infiltration or lesions. Significant increases in IL-1β and TNF-α levels were also found in *L*. *lactis*-inoculated glands. The above findings seem to suggest that food-grade *L*. *lactis* at a high-inoculum dose such as an overnight culture may elicit a suppurative inflammatory response in the mammary gland, thus becoming a potential mastitis-causing pathogen. Because of the unpredictable potential of *L*. *lactis* in acting as a potential mastitis pathogen, this organism cannot be considered a safe treatment for bovine mastitis.

## Introduction

Mastitis, generally defined as the inflammation of the mammary gland, is one of the most prevalent diseases affecting dairy ruminants worldwide [1,2]. This disease causes huge economic losses due to decreases in milk quantity and quality, veterinary services and labour expenses [3].

Antibiotics are extensively used for the treatment and prevention of mastitis in dairy ruminants [4]. Due to concerns regarding the growing antibiotic resistance of mastitis-causing pathogens that often make antibiotic therapy ineffective and the implications of the overuse of antibiotics on public health, the development of alternatives are strongly encouraged.

The use of bacteriocins, such as nisin and lacticin 3147, has recently been proposed as an alternative to antibiotics for treating and preventing bovine mastitis. The efficacy of bacteriocin-based formulations administered to the teat by dipping or intramammary infusion has been documented both for treatment of clinical and subclinical bovine mastitis [5,6] and prevention of streptococcal [7] and staphylococcal [8] mastitis infections in cattle. However, controversial results are described in literature regarding the use of lactic acid bacteria (LAB) as intramammary inocula for treating mastitis in dairy ruminants. On the one hand, safety and efficacy in reducing *Staphylococcus aureus* infection have been described with the inoculation of the live culture of *Lactococcus lactis* DPC 3147 [9]. On the other hand, inefficacy of the intramammary *Lactobacillus* therapy in decreasing coagulase-negative staphylococci (CNS) infections in cattle, has been documented [10]. Recently, our group has conducted *in vitro* investigations of the probiotic potential of *L*. *lactis* subsp. *lactis* LMG 7930, a food-grade nisin-producing strain, against mastitis-causing pathogens [11]. Our *in vitro* results suggested that this strain might be a promising candidate for the development of new strategies of mastitis control in ruminants [11]. We then discussed the efficacy and safety of the intramammary infusion of an overnight culture of *L*. *lactis* LMG 7930 in *in vivo* studies under field conditions (still unpublished data from our group). We conducted two trials involving naturally infected ewes with clinical and subclinical staphylococcal mastitis. We found that the *L*. *lactis* treatment rapidly activated the mammary glands' innate immune response and initiated an inflammatory response as evidenced by the recruitment of polymorphonuclear neutrophils (PMNs) and increased somatic cell counts. But while leading to a transient clearance of CNS in the gland, this response caused mild to moderate clinical cases of mastitis characterized by abnormal milk secretions and udder inflammation (still unpublished data from our group).

To further investigate the host immune response induced by the intramammary infusion of the *L*. *lactis* culture, we used the mouse mastitis model that is known to be more time- and cost-effective than larger animal models. The experimental mouse model of mastitis was developed and characterized by Chandler in 1970 [12]. Although differences may exist between ruminants and murine mammary glands [13], the mouse model of *S*. *aureus* mastitis provided reliable, useful and economical alternatives for studying bacterial pathogenesis of bovine mastitis-causing pathogens [14–17] and for primary evaluation of experimental drugs [18]. Recently, a mouse model of *S*. *chromogenes* mastitis has been proposed by Breyne and colleagues to study differences between bovine associated CNS species [19].

In this study, we have explored the effects of the intramammary inoculum of an overnight culture of the food-grade *L*. *lactis* LMG 7930 strain on the mouse mammary gland immune response by proposing, for the first time, to the best of our knowledge, a mouse model of *L*. *lactis* mastitis. Inoculum size, time of infection, bacterial recovery from the gland, histological features and proinflammatory cytokine production are described.

## Material and methods

### Bacterial strains

*L*. *lactis* subsp. *lactis* LMG 7930 (BCCM/LMG Bacteria Collection, Belgium), a food grade strain used in the dairy industry in the production of Swiss cheese, was used for the intramammary inoculum. Two mastitis-causing pathogens, *S*. *aureus* LMG 16805 isolated from bovine mastitis (BCCM/LMG Bacteria Collection, Belgium) and *S*. *chromogenes* 100-SA isolated from a chronic case of subclinical mastitis in sheep (Istituto Zooprofilattico Sperimentale della Sardegna Bacteria Collection, Italy), were also used in this study.

Bacterial cultures were performed in Trypticase Soy Broth (TSB, BD, Italy) and incubated at 37°C for 24 h. Overnight cultures were used for the intramammary injection in mice. *L*. *lactis* was used as is, while *S*. *chromogenes* and *S*. *aureus* were properly diluted in PSB to achieve the desired concentration (see the “Mouse mastitis model of infection” section below). The bacterial inoculum size was determined by serially diluting the culture and pour-plating the dilutions on TSB agar.

### Mice

A total of 41 timed-pregnant CD-1 mice (ca. 17 days of gestation), purchased from Envigo RMS Srl (Italy), were considered. Mice were randomly divided into six groups: LL (n = 11, treated with *L*. *lactis*), S.CH (n = 7, treated with *S*. *chromogenes*), S.CH-LL (n = 7, treated with *S*. *chromogenes* and *L*. *lactis*), S.AU (n = 3, treated with *S*. *aureus*), PBS (n = 6, control group treated with sterile PBS solution) and UNT (n = 7, untreated control group).

The animals were individually housed in the animal facilities of the Istituto Superiore di Sanità (Rome, Italy). All procedures involving animals were approved by the Italian Ministry of Health (Permit number 120/2014-B-24/03/2014). Animal care and treatment were conducted in accordance with the institutional guidelines and international laws and policies (Directive 2010/63/EU on the protection of animals used for scientific purposes).

### Mouse mastitis model of infection

The general procedure for mouse mammary gland infection was adapted from the method used by Breyne *et al*. [19] and Brouillette *et al*. [18]. Briefly, CD-1 lactating mice were used 10–12 days after birth of the offspring. The pups were removed 2 h before inoculation into the teat canal of the inguinal mammary glands L5 (on the left) and R5 (on the right). One hundred μl of either an overnight culture of *L*. *lactis* (approximately 10^8^ CFU/ml) or PBS-diluted cultures of *S*. *chromogenes* (approximately 10^6^ CFU/ml) and *S*. *aureus* (approximately 10^3^ CFU/ml) were used for the intramammary injections in groups LL, S.CH and S.AU, respectively. In the S.CH-LL group, each gland received two consecutive injections: an initial inoculum of 100 μl of *S*. *chromogenes* (approximately 10^6^ CFU/ml) was followed by a second inoculum of 100 μl of *L*. *lactis* (approximately 10^8^ CFU/ml), 4 h later, in order to facilitate absorption. In the PBS control group the inguinal glands were treated with 100 μl of PBS (Sigma Aldrich Srl, Italy). Syringes with 33-gauge blunt needles were used for the inocula.

Twenty-four hours post infection (p.i.), all mice were sacrificed by cervical dislocation. For each animal, both L5 and R5 mammary glands were aseptically collected and pooled for cultures and histological investigations. Cytokine production was also measured in the LL, PBS and UNT groups.

### Microbiological culture of mammary glands

Inguinal mammary gland samples from each animal were weighed, homogenized and cultured. In summary, tissue samples were homogenized (1:1) in PBS and then serially diluted (1:10). Dilutions were plated onto both Trypticase Soy Agar (TSA, BD, Italy)–nonselective medium–and Mannitol Salt Agar (MSA, BD, Italy)–selective and differential medium used for the isolation of pathogenic staphylococci–to enumerate bacteria. PBS and UNT control glands were also plated onto both TSA and MRA. Plates were incubated for 24–48 h at 37°C until colonies became visible for enumeration. In S.CH-LL, *L*. *lactis* counts were determined by subtracting the total *S*. *chromogenes* + *L*. *lactis* obtained on TSA from the *S*. *chromogens* counts obtained on MSA.

Random colonies were picked from each plate and molecularly identified by PCR amplifying and sequencing 16S rDNA [20]. Results were expressed as colony forming unit per gram (CFU/g) of mammary gland. Mean values per group with standard deviation were then calculated.

### Histological investigation

Mammary gland samples from all groups were fixed in 10% neutral buffered formalin and routinely processed to obtain 4 μm paraffin-embedded histological sections which were then stained with haematoxylin and eosin and examined via light microscopy.

Lesions observed were measured semiquantitatively using a scoring system based on the characterization of the inflammatory cell infiltrate, specifically by accumulation of PMNs, according to the literature [21]. A scale from 1 to 4 was applied to the following alterations: 0, no lesions, absence of interstitial and/or alveolar inflammatory cell infiltrate and undamaged tissue; 1, focal to multifocal, mild interstitial and/or alveolar infiltrate of inflammatory cells and undamaged tissue; 2, multifocal, moderate interstitial and/or alveolar infiltrate of inflammatory cells and undamaged tissue; 3, severe, diffuse interstitial and/or alveolar infiltrate of inflammatory cells and focal areas of tissue damage; 4, severe, diffuse interstitial and/or alveolar infiltrate of inflammatory cells and extensive necrotic areas. All the slides were assessed blinded by three observers (EB, LS and MTC) using a light microscope, and the discordant cases were reviewed at a multi-head microscope until a consensus was reached.

### Cytokine analysis

Mammary gland samples from the LL, PBS and UNT groups were weighed and homogenized in PBS (1: 9, w/v) supplemented with a cocktail of protease inhibitors which is able to inhibit serine and cysteine proteases, calpain proteases and metallo-proteases (Roche, Germany). The homogenate was centrifuged at 2000 x g for 30 min at 4°C, and the supernatant was collected. The levels of the cytokines interleukin (IL)-1β and tumour necrosis factor (TNF)-α were determined by using an ELISA kit (PeproTech, Germany), according to the manufacturer’s instructions and the literature [22]. ELISA tests were performed in triplicate. Mean values per group with standard deviation were then calculated.

### Statistical analysis

Mean values per group with standard deviation were used to describe data sets of bacterial recovery and cytokine production.

Two-way ANOVA was performed to analyse main effects of *L*. *lactis* and *S*. *chromogenes* inoculations, as well as their interaction. Post-hoc Tukey’s multiple comparisons test was performed where appropriate.

Inter-group analyses of differences in TNF-α and IL-1β levels among LL, PBS and UNT were performed by the one-way ANOVA, followed by Tukey’s multiple comparisons test where applicable.

GraphPad Prism 6 version 6.07 for Windows, GraphPad Software, La Jolla California USA, www.graphpad.com, was used. Differences were considered significant at *P*< 0.05.

## Results

### Recovery of bacteria from glands

**Fig 1:**
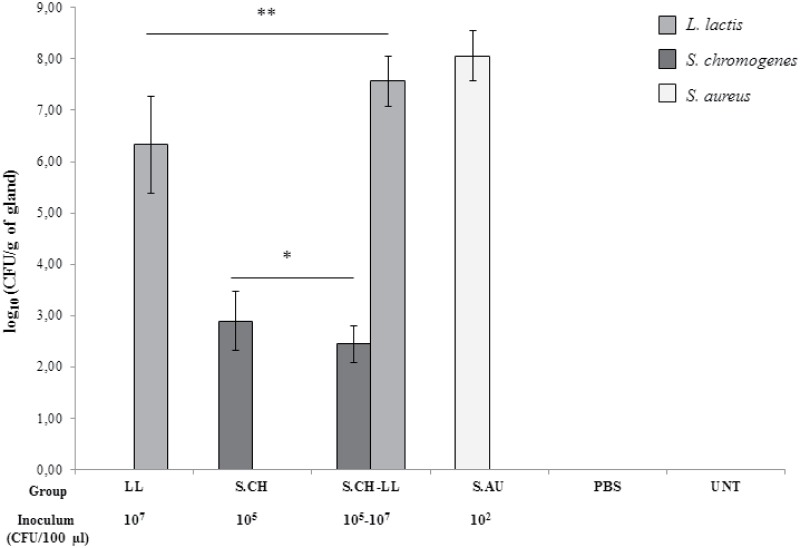
*L*. *lactis*, *S*. *chromogenes* and *S*. *aureus* counts. All inoculated bacteria were counted in mammary glands at 24 h p.i. in treated (LL, S.CH, S.CH-LL, S.AU and PBS) and untreated (UNT) groups. Results are expressed as mean values with standard deviation of log_10_ CFU/g values. The initial bacterial inoculum size is shown. Significant differences are indicated. * *P* ≤ 0.05, ** *P* ≤ 0.01.

Bacterial count mean values of *L*. *lactis* (groups LL and S.CH-LL), *S*. *chromogenes* (groups S.CH and S.CH-LL) and *S*. *aureus* (S.AU group) are shown in Fig 1 and expressed as log_10_ CFU/g of gland.

All bacteria here tested were recovered from the glands at 24 h p.i. The mean value of *S*. *aureus* count in S.AU (1.79 x 10^8^ CFU/g of gland) was greater than the mean values recorded for *L*. *lactis* in LL (9.32 x 10^6^ CFU/g of gland) and S.CH-LL (5.78 x 10^7^ CFU/g of gland) and for *S*. *chromogenes* in S.CH (1.55 x 10^3^ CFU/g of gland) and S.CH-LL (2.56 x 10^2^ CFU/g of gland) even though the inoculum size of *S*. *aureus* was smaller than that of *L*. *lactis* or *S*. *chromogenes*. Main effects of *S*. *chromogenes* (F = 6.80, *P* = 0.0147), *L*. *lactis* (F = 13.03, *P* = 0.0012) as well as their interaction (F = 6.80, *P* = 0.0147) were observed on *L*. *lactis* counts. A significant increase of *L*. *lactis* recovery was found in S.CH-LL compared to LL (*P* = 0.0025). Main effects of *S*. *chromogenes* (F = 8.50, *P* = 0.0071), *L*. *lactis* (F = 4.36, *P* = 0.0464) and their interaction (F = 4.36, *P* = 0.0464) were also observed on *S*. *chromogenes* counts. A significantly lower bacteria recovery was recorded in S.CH-LL mice compared to the S.CH group (*P* = 0.0364). No bacterial colonization was observed in the control glands (groups PBS and UNT).

### Histopathological changes

**Fig 2:**
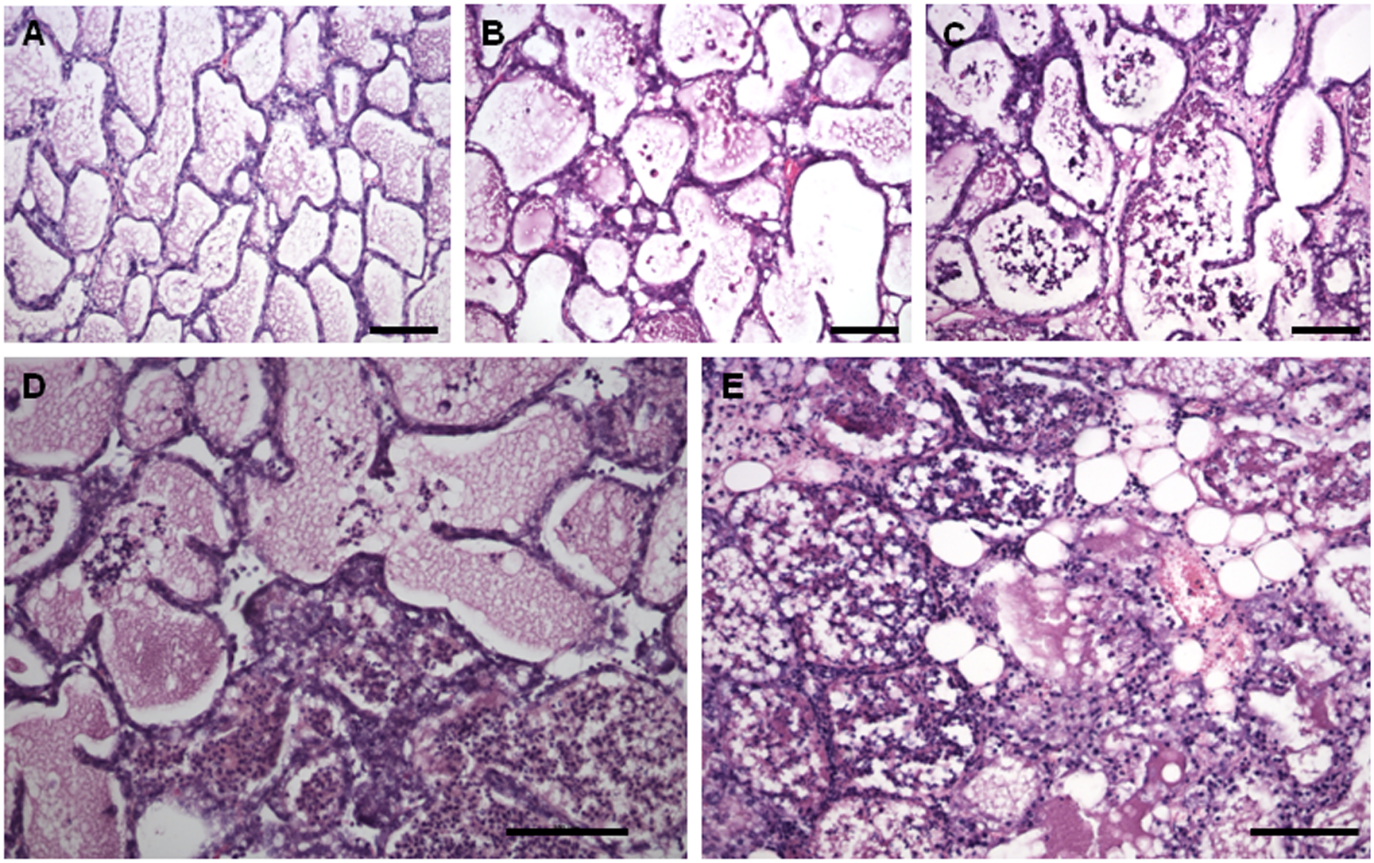
Histological changes in the murine mammary glands. Panels A: UNT group, score 0, no infiltration and undamaged tissue; B: LL group, score 1, mild PMN infiltration in isolated areas of tissue sections; C: LL group, score 2, moderate PMN alveolar and interstitial infiltration; D: LL group, score 3, severe PMN infiltration and focal areas of tissue damage; E: S.AU group, score 4, severe PMN infiltration and extensive necrotic areas. Magnifications 20X, bar = 100 μm.

Representative histological sections, scored from 0 to 4, are shown in Fig 2. Most of the mammary gland tissue samples from the PBS (4/6) and UNT (5/7) groups, as well as from S.CH (6/7), showed a normal structure and no infiltration or lesions (score 0), as shown in panel A. The rest showed minimal interstitial non suppurative inflammations (score 1): 2/6 in PBS, 2/7 in UNT and 1/6 in S.CH.

The glands challenged with *L*. *lactis* (LL and S.CH-LL) showed variable histological features, with scores ranging from 0 to 3. In LL, tissue damage recorded ranged from no lesions (3/11, score 0) to mild (1/11), moderate (5/11) and severe (2/11) inflammatory cell infiltrations, scored from 1 to 3, respectively (panels B-D). In S.CH-LL, no lesions (1/7, score 0), as well as mild (2/7, score 1) and severe inflammatory cell infiltrations (4/7, score 3) were histologically documented. In S.AU, the *S*. *aureus* challenge caused severe PMN infiltrations with extensive necrotic areas (3/3, score 4) in the mammary glands (panel E).

### Effect of *L*. *lactis* on the proinflammatory cytokine production

TNF-α and IL-1β levels were measured in *L*. *lactis*-treated (LL), PBS-treated (PBS) and untreated (UNT) control glands at 24 h p.i. and expressed as mean values with standard deviations (pg/ml) in Table 1.

**Table 1:** Proinflammatory cytokine production in the LL, PBS and UNT groups.

Cytokine (pg/ml)	LL	PBS	UNT	Significant difference
TNF-α	267 ± 83	165 ± 49	142 ± 62	LL *vs* UNT*P =* 0.0351
IL-1β	5383 ± 1007	4117 ± 300	3762 ± 534	LL *vs* UNT*P =* 0.0204

Results are expressed as mean values with standard deviation. Differences in TNF-α and IL-1β levels among LL, PBS and UNT were determined by the one-way ANOVA, followed by Tukey’s multiple comparisons test where applicable. Significant differences (*P*<0.05) are shown.

Significant differences in the proinflammatory cytokine production data set were observed comparing LL *versus* UNT only. Significant increases in TNF-α (*P =* 0.0351) and lL-1β (*P =* 0.0204) levels were recorded in LL. Comparing LL *versus* PBS data sets, no statistical differences were found, although higher levels of TNF-α and IL-1β were recorded in LL. Higher levels of TNF-α and IL-1β were recorded in two *L*. *lactis*-treated glands presenting severe PMN infiltrations at histology. The more pronounced dispersion was observed in IL-1β values in LL as measured by the standard deviation. Results as shown in Table 1.

## Discussion

We have previously assessed, in two trials, the efficacy and safety of intramammary infusion of a live overnight culture of the food-grade *Lactococcus lactis* LMG 7930 for the treatment of subclinical (by CNS infections) and clinical (by *S*. *aureus* infections) mastitis in lactating ewes. We have found that, under our experimental conditions, the live bacterial culture rapidly activated the mammary innate immune system. This led to a transient clearance of the CNS in the gland, but also caused mild to moderate clinical cases of mastitis (still unpublished data from our group). To make further progress in our understanding of *L*. *lactis* mastitis, we decided to study the interaction between *L*. *lactis* LMG 7930 and the mammary gland in a mouse mastitis model in greater detail.

The mouse mastitis model has been proven to be a fast, cheap and reproducible intermediate *in vivo* alternative for studying the specific host immune response to intramammary injection with major mastitis pathogens, such as *Escherichia coli* and *S*. *aureus* [15,23]. Recently, a pilot study has suggested that a mouse mastitis CNS model can mimic bovine CNS mastitis and be used to study differences between bovine CNS species and strains [19]. Here we investigated whether an intraductal injection of an overnight *L*. *lactis* culture into the murine mammary gland (approximately 10^7^ CFU/100 μl) can be used to evaluate *L*. *lactis* survival in the tissue and success in overcoming host immune response 24 h p.i. Therefore we conducted microbial cultures and histopathological investigations on *L*.*lactis*-treated mammary glands. We also treated additional mice with bacterial suspensions of mastitis-causing pathogens, i.e. *S*. *chromogenes* (S.CH) or *S*. *aureus* (S.AU) in order to have two mouse mastitis models as complementary tools to better evaluate the effects of the *L*. *lactis* challenge into the mammary gland. We decided to inoculate approximately 10^5^ CFU/100 μl of *S*. *chromogenes* because a previous study had documented that an *S*. *chromogenes* inoculum dose as high as 10^5^ CFU displayed clinical symptoms of inflammation if inoculated into murine mammary glands [19]. According to the validated mouse mastitis *S*. *aureus* model, we inoculated approximately 10^2^ CFU/100 μl of *S*. *aureus* because an inoculum dose as high as 100 CFU of that pathogen has been proven to be nonfatal and to cause a significant level of infection with evident signs of mammary gland inflammation at 24 h p.i. [18,19]. An additional group was included in our study: S.CH-LL, containing animals treated with two separate injections–*S*. *chromogenes* first, followed by *L*. *lactis*–in order to assess whether *L*. *lactis* was able to affect *S*. *chromogenes* survival in the mammary gland.

Microbiological results showed that *L*. *lactis* was able to survive in the mammary gland 24 h p.i., as were the mastitis pathogens *S*. *chromogenes* and *S*. *aureus*. In contrast, no bacterial colonization was observed in the control glands (groups PBS and UNT). We observed that *L*. *lactis* reduced *S*. *chromogenes* survival in the glands in S.CH-LL at 24 h p.i., and increased its own survival ability by coexisting with the pathogen. It seems that *L*. *lactis* and *S*. *chromogenes* engaged in interspecies cooperative behaviours rather than competitive interactions. Bacterial species may in fact coexist with, or dominate, other organisms, competing for the same pool of resources by adopting many active mechanisms [24]. *L*. *lactis*-treated glands showed variable histological features, ranging from undamaged tissue with no inflammatory cell infiltrate to severe PMN infiltrate with focal areas of tissue damage (scores 0 to 3). A moderate mammary gland inflammation characterized by alveolar and/or interstitial PMN infiltration was the most prevalent change observed in the mammary glands challenged with *L*. *lactis* alone (LL). Glands challenged with both *S*. *chromogenes* and *L*. *lactis* (S.CH-LL), however, showed a more severe degree of inflammation. Neutrophil infiltration was observed in almost all glands and in both interstitium and alveoli, suggesting a more severe inflammation than observed in udders challenged with *L*. *lactis* alone. These differences in histology are likely related to the greater number of *L*. *lactis* colonies measured in S.CH-LL than that recorded in LL.

Proinflammatory cytokine production was measured in LL, PBS and UNT glands. Significant increases in IL-1β and TNF-α levels were found in *L*. *lactis*-inoculated glands by comparing LL with UNT. However, no significant changes were observed between groups LL and PBS, although we recorded higher proinflammatory cytokine trends and a more pronounced dispersion of values in LL. The cytokine production results are in accordance with the greater number of glands with inflammatory cells infiltrate that we recorded in LL compared with the control groups.

Predictably, bacterial growth in *S*. *aureus* inoculated glands was the highest we recorded, even though the inoculum size was the smallest. Moreover, *S*. *aureus*-treated glands all showed the most severe histological grade of inflammation (score 4). Both results are compatible with previous studies [18,19] and seem to support an inoculum dose as high as 100 CFU of a mastitis *S*. *aureus* isolate per gland, irrespective of the strain, like the injection that was able to cause severe mammary gland inflammation in the mouse model at 24 h p.i. That confirms lactating mice as animals very susceptible to intramammary infusion by *S*. *aureus*. Contrary to our expectations, the mastitis isolate of *S*. *chromogenes* here used was unable to induce mastitis when it was used alone at the inoculum size as high as 10^5^ CFU at 24 h p.i. Indeed, most mammary glands in S.CH showed normal structures with no inflammatory infiltration or lesions detected by the histological exam. Concordant results were found in the control groups (PBS and UNT). Few cases of mammary gland inflammation–and, moreover, mild ones–were recorded in S.CH, as well as in PBS and UNT. That mild inflammation seemed to have been caused by milk accumulation (physiological inflammation) rather than by the pathogen or inoculum itself. The rapid accumulation of milk in the mammary gland–due to the permanent removal of offspring from a lactating mother, which is referred to in the literature as “forced weaning”–has been documented to result in elevation of inflammatory mediators [25] and influx of macrophages into the mammary gland [26,27], all of which are critical components of the cell death and tissue remodelling process [28]. Our results together with the literature [19] confirm that CNSs are minor mastitis pathogens and that epidemiologically different mastitis isolates of CNS strains may induce variable host innate immune responses in the mouse mammary gland.

In conclusion, the *L*. *lactis* inoculum caused variable histological features in the mammary gland, ranging from undamaged tissue to severe inflammation. Our results suggest that bacteria with GRAS status, such as *L*. *lactis*, can act as mastitis-causing pathogens when inoculated at high density into the mammary glands of lactating mice. Because of the unpredictable potential of *L*. *lactis* in acting as a potential mastitis pathogen, this organism cannot be considered a safe treatment for bovine mastitis.
